# Supplementary material for: Evaluation of Biomarkers of Severity in Patients with COVID-19 Infection
Source: J Clin Med. 2021 Aug 24;10(17):3775. doi: 10.3390/jcm10173775 (PMC8432011; doi:10.3390/jcm10173775)
Supplement: Supplementary file 1 [file jcm-10-03775-s001.zip › jcm-1339111-supplementary.pdf]

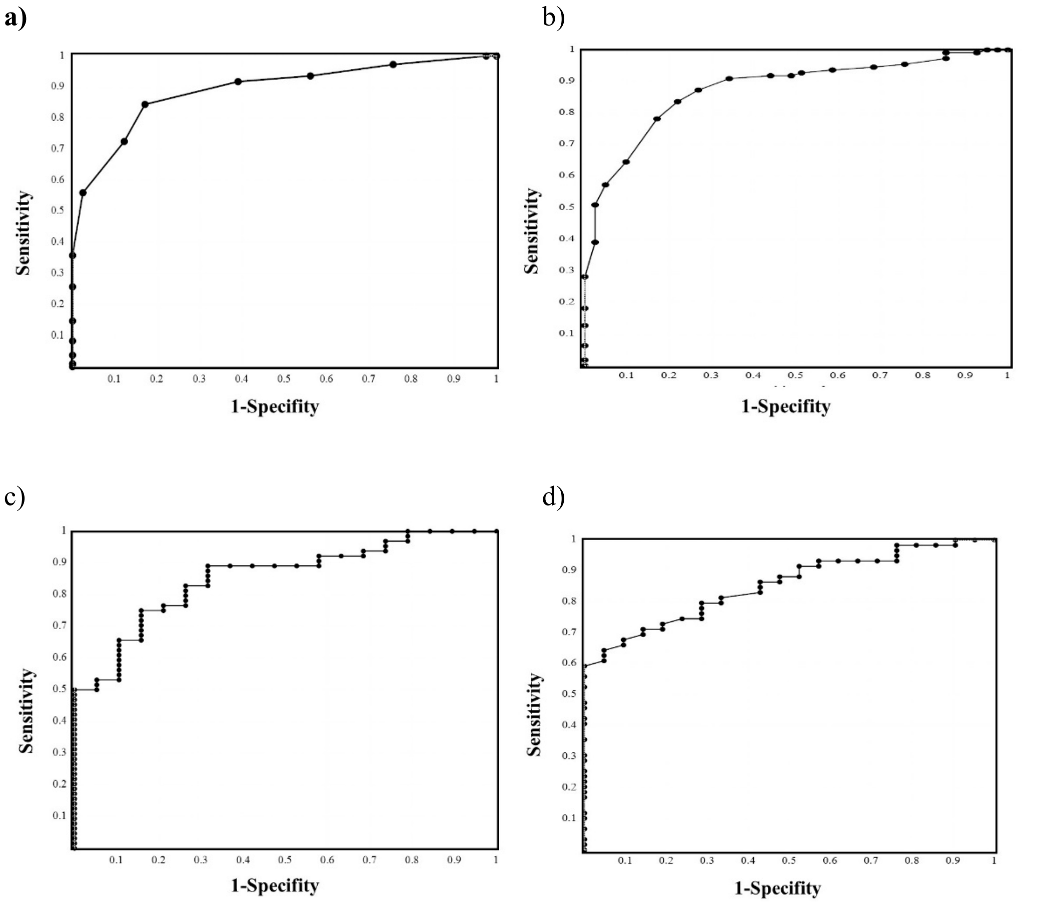

Supplementary Figure S1 (Figure S1) An ROC analysis to determine the cutoff values of the A/G ratio (a), albumin (b), ferritin (c) and procalcitonin (d) (mild to moderate I vs. ≥moderate II to severe)
